# Increased difficulty and complications of delayed laparoscopic cholecystectomy following percutaneous transhepatic gallbladder drainage in acute cholecystitis: a retrospective study

**DOI:** 10.1186/s12893-023-02185-2

**Published:** 2023-09-13

**Authors:** Ya-qi Liu, Xuan Cai, Zhi-xue Zheng, Fang-jingwei Xu, Jing-tao Bi

**Affiliations:** 1 Department of General Surgery, Beijing Jishuitan Hospital, Capital Medical University, Beijing, 100035 China; 2 Department of Research and Development, China National Biotec Group, Beijing, 100029 China

**Keywords:** Percutaneous transhepatic gallbladder drainage, Delayed laparoscopic cholecystectomy, Acute cholecystitis, Morbidity rate, Retrospective study

## Abstract

**Background:**

Percutaneous transhepatic gallbladder drainage (PTGBD) is a relatively less invasive alternative treatment to cholecystostomy. However, the influence of the difficulty of delayed laparoscopic cholecystectomy (DLC) after PTGBD on clinical outcomes remains unknown. This study aimed to evaluate the clinical effects of DLC following PTGBD.

**Methods:**

The clinical data of 113 patients diagnosed with moderate (grade II) acute cholecystitis according to the 2018 Tokyo Guidelines in the acute phase and who underwent DLC in our hospital from January 2018 to February 2022 were retrospectively collected and separated into two groups according to whether they received PTGBD treatment in the acute stage. The PTGBD group comprised 27 cases, and the no-PTGBD group included 86 cases. The TG18 difficulty score was used to evaluate every surgical procedure in the cases by reviewing the surgical videos. The clinical baseline characteristics and post-treatment outcomes were also evaluated.

**Results:**

Both groups showed significant differences in length of postoperative stay, blood loss, operation time, and difficulty score. The PTGBD group showed a significantly longer postoperative stay and operation time, more blood loss, and a much higher difficulty score than the no-PTGBD group. Conversion rates did not differ. The morbidity rate in the PTGBD group was statistically higher.

**Conclusions:**

PTGBD is an efficient way to relieve the symptoms of acute cholecystitis. However, it may increase the difficulty and complications of DLC.

## Background

A consensus on managing acute cholecystitis (AC) has been reached in the Tokyo Guidelines 2018 (TG18) [1]. TG18 established that the appropriate treatment for AC was selected based on AC severity grading. A personalized treatment strategy was developed according to the severity of the inflammation.

As an alternative treatment to cholecystostomy, percutaneous transhepatic gallbladder drainage (PTGBD) is performed for moderate-to-severe cases with more complications and less effective antibiotic therapy [2] Several studies [3–8] have described PTGBD as being less invasive and having a lower risk of adverse events than cholecystostomy. However, the optimal timing of tube removal has not yet been confirmed [2]. Thus, surgical interval after PTGBD remains controversial. PTGBD complications such as tube plugging, unplanned tube removal, and bile leak are sometimes unavoidable.

The effect of delayed laparoscopic cholecystectomy (DLC) after PTGBD remains unknown. Fibrinous exudate and adhesion can be present in PTGBD, which aggravates AC adhesion. However, it can relieve tension in the gallbladder and reduce inflammation. Further investigation is needed to determine whether PTGBD positively or negatively affects surgical difficulty in DLC cases.

This study compared the outcomes of DLC after PTGBD with DLC without PTGBD using the TG18 score system [9] (Table 1) by reviewing videos of surgical procedures. Clinical characteristics and post-treatment outcomes were also evaluated.

**Table 1:** Surgical difficulty grading system for laparoscopic cholecystectomy

	Surgical difficulty score						
	0	1	2	3	4	5	6
Fibrosis /scarring of the gallbladder							
Around the gallbladder	No findings		Fibrotic adhesion or partial scarring		Diffuse scarring		
Calot’s triangle area	No findings		Sparse fibrosis	Dense fibrosis	Partial scarring	Diffuse scarring	
Gallbladder bed	No findings	Sparse fibrosis	Dense fibrosis	Partial scarring	Diffuse scarring		
Additional findings of the gallbladder and its surroundings	No findings	Edematous change		Easy bleeding	Necrotic changes	Cholecystoenteric fistula	Cholecystocholedochal fistula (Mirizzi syndrome)
				Perforated gallbladder wall and/or abscess formation	Abscess formation toward the liver parenchyma	Impacted gallstone in the confluence (Mirizzi syndrome)	
Intra-abdominal factors unrelated to inflammation	No findings	Non-inflammatory adhesion	Excessive visceral fat	GB neck mounting on the common bile duct	Inversion of the GB or collateral vein formation due to liver cirrhosis		
				Anomalous bile duct			

## Methods

### Patient selection

**Fig. 1:**
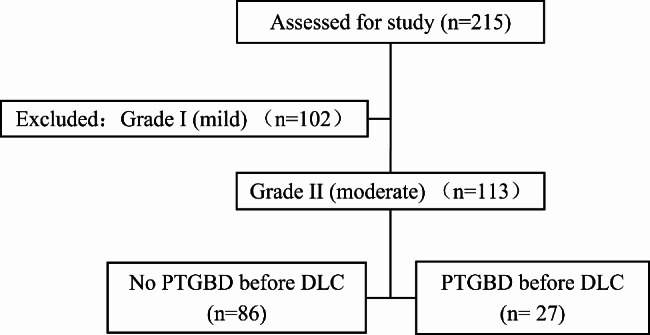
Flowchart illustrating the patient population. A total of 215 patients who were diagnosed with AC and underwent DLC were assessed. Patients diagnosed with Grade I (mild) AC were excluded. The remaining 113 patients were all classified as Grade II (moderate) and divided into two groups based on whether they received the PTGBD treatment in the acute phase. AC, acute cholecystitis

This retrospective study was performed between January 2018 and February 2022. A total of 215 patients diagnosed with AC who received DLC were assessed. They were classified by severity grade according to TG18. Patients with Grade I (mild) AC were excluded. The remaining 113 patients were classified as Grade II (moderate), which were associated with any one of the following conditions: elevated WBC count (> 18,000/mm^3^), palpable tender mass in the right upper abdominal quadrant, duration of complaints > 72 h, and marked local inflammation (gangrenous cholecystitis, pericholecystic abscess, hepatic abscess, biliary peritonitis, and emphysematous cholecystitis). The patients with shock requiring vasopressin, or with severe dysfunction of other organs who are classified as Grade III (severe), were not found. The patients with choledocholithiasis, who need CBD exploration, intraoperative choledochoscopy, or cholangiography were excluded in this study. The 113 patients of Grade II AC divided into two groups based on whether they received PTGBD treatment in the acute phase (Fig. 1).

### Data collection

A PTGBD tube (8Fr pigtail) was placed in the gallbladder of all the patients in the PTGBD group. In the acute phase, patients were discharged with tube approximately 1 week after PTGBD insertion. Antibiotics were discontinued after discharge. Before surgery, the patients were reviewed at the outpatient clinic. Moreover, the tube remained in place until the DLC was performed. We performed DLC after at least 8 weeks following PTGBD.

The measured variables were the length of postoperative hospital stay, operation time, blood loss, conversion rate, and morbidity rate. The morbidity scoring system [10] was used to analyze the severity of complications. By reviewing the operative videos, we used the difficulty score for intraoperative findings from TG18 [9] to evaluate every surgical procedure.

### Statistical analysis

Data processing and statistical analyses were performed using SPSS 22.0 statistical analysis package. Normally distributed data were analyzed using the *t*-test; otherwise, the Mann–Whitney *U* test was used. The chi-squared test was performed on the counted data. *P* < 0.050 was considered statistically significant.

## Results

A total of 113 patients with Grade II (moderate) AC underwent DLC. The clinical characteristics of the patients at the time of DLC are summarized in Table 2. Of the 113 patients, 86 underwent DLC without prior PTGBD (the no-PTGBD group), and 27 underwent PTGBD prior to DLC (the PTGBD group).

**Table 2:** Baseline characteristics comparing patients in the no-PTGBD vs. PTGBD groups at the time of delayed laparoscopic cholecystectomy

	No-PTGBD group (n = 86)	PTGBD group (n = 27)	*P-*value
Sex			
Male (%)	44 (51.2)	14 (51.9)	0.950
Female (%)	42 (48.8)	13 (48.1)	
Age	53.07 ± 14.10	60.52 ± 13.76	0.019*
BMI	25.41 ± 3.59	24.92 ± 2.80	0.467
CCI (%)			
0	62 (72.1)	16 (59.3)	0.191
1	19 (22.1)	11 (40.7)	
2	3 (3.5)	0	
≥3	2 (2.3)	0	
ASA-PS (%)			
1	47 (54.7)	9 (33.3)	0.153
2	32 (37.2)	15 (55.6)	
≥3	7 (8.1)	3 (11.1)	

ASA-PS, American Society of Anesthesiologists physical status; CCI, Charlson comorbidity index. *P < 0.05 for the between-group comparison

The variables and surgical outcomes of the PTGBD group were compared with those of the no-PTGBD group. The PTGBD group showed a significantly longer postoperative hospital stay (median 3 d vs. 2 d) and operation time (median 98 min vs. 46 min), more blood loss (median 50 mL vs. 20 mL), and much higher difficulty score (median 4 points vs. 2 points) than the no-PTGBD group (*P* < 0.05) (Table 3). The conversion rate was approximately the same in both groups (*P* = 0.382). The PTGBD group also showed a higher morbidity rate (25.9% vs. 7.0%) than the no-PTGBD group (*P* = 0.007) (Table 4).

**Table 3:** Surgical outcome comparisons between the PTGBD and no-PTGBD groups (1)

Variables	No-PTGBD group (n = 86)		PTGBD group (n = 27)		*Z*	*P-*value
	Median	Mean rank	Median	Mean rank		
Postoperative hospital stay (d)	2	50.49	3	77.74	-3.865	0.000^*^
Operation time (min)	46	45.78	98	92.74	-6.499	0.000^*^
Blood loss (mL)	20	51.70	50	73.87	-3.202	0.001^*^
Difficulty score	2	48.09	4	85.37	-5.336	0.000^*^

*P < 0.05 for the between-group comparison

**Table 4:** Surgical outcomes comparisons between the PTGBD and no-PTGBD groups (2)

	No-PTGBD group (n = 86)		PTGBD group (n = 27)	x^2^	*P-*value
Conversion		1 (1.2%)	1 (3.7%)	0.763	0.382
Morbidity		6 (7.0%)	7 (25.9%)	7.247	0.007*

*P < 0.05 for the between-group comparison

The morbidity scoring system was used to analyze the severity of complications. Table 5 shows the rates of complications in both groups. The rate of acute pancreatitis after surgery was the main difference in complications between the two groups. Pancreatitis occurred in two cases in the PTGBD group, whereas the no-PTGBD group had no cases.

**Table 5:** Morbidity rates for morbidity score items

Complications	Score points	No-PTGBD group (n = 86)	PTGBD group (n = 27)	x^2^	*P*-value
Persistent abdominal pain	1	1 (1.16%)	1 (3.70%)	0.763	0.382
Persistent fever	1	2 (2.33%)	1 (3.70%)	0.151	0.698
Persistently raised signs of infection	1	0	1 (3.70%)	3.214	0.073
Wound-healing complication	2	1 (1.16%)	1 (3.70%)	0.763	0.382
Thrombosis	3	0	0	/	/
Bleeding	3	0	0	/	/
Cholangitis	3	1 (1.16%)	0	0.317	0.574
Icterus	3	1 (1.16%)	0		
Bile leakage	3	0	1 (3.70%)	3.214	0.073
Abscess	3	0	1 (3.70%)	3.214	0.073
Pneumonia	3	0	1 (3.70%)	3.214	0.073
Embolic lung disease	4	0	0	/	/
Peritonitis	4	0	0	/	/
Pancreatitis	4	0	2 (7.41%)	6.485	0.011*
Renal failure	4	0	0	/	/
Relaparotomy	5	0	0	/	/
Cerebral ischemia or bleeding	5	0	0	/	/
Myocardial infarction	5	0	0	/	/
Septic shock	5	0	0	/	/
Death	63	0	0	/	/

*P < 0.05 for the between-group comparison

## Discussion

This study assessed the extent of DLC difficulty after PTGBD. PTGBD has been established as an effective method for gallbladder drainage to eliminate obstructive symptoms [11, 12]. Percutaneous transhepatic puncture was the primary procedure. A tube apterium exists between the liver and the abdominal wall. The formation of the fibrin sinus tract determines the timing of tube removal. Notably, early tube removal can lead to bile leaks. However, long-term catheterization may generate more fibrinous exudate and adhesions, which can increase the difficulty of surgery. In our study, we observed that DLC after PTGBD required a significantly longer postoperative hospital stay and operation time and involved more blood loss and a higher difficulty score than DLC without prior PTGBD. The Tokyo guidelines clearly state that there is currently no high-quality scientific evidence on the optimal surgical timing for LC after PTGBD. A study has suggested that PTGBD with advanced LC has a long time of catheter placement, and the incidence of complications, such as catheter detachment, blockage, and displacement, increases significantly, which affect the quality of life of patients [13]. Another study suggested that drainage tube removal is safe and effective when performed after a short drainage period of 7–10 d [14]. In a study [15] of 6145 patients who underwent LC after PTGBD, the results showed that the complications of LC surgery increased within 1 month after PTGBD and the incidence of PTGBD-related complications such as catheter detachment, blockage and displacement increased clearly after 8 weeks of PTGBD, thus confirming that the best time for LC was 4–8 weeks after PTGBD.

The severity of calculus incarceration can be another factor that affects the difficulty of surgery. Gallbladder pressure was decreased by PTGBD during the acute phase in AC patients, but calculus incarceration was not resolved. Fibrotic and scarring adhesions were consistently observed. Additionally, we observed that the intraoperative finding score for “Appearance of the Calot’s triangle area” could reach 5 points, indicating that serious fibrotic change or scarring in the Calot’s triangle persisted even after 8 weeks following PTGBD. This provides evidence supporting the importance of early laparoscopic cholecystectomy in AC cases, as suggested by TG18. However, in China, the population base is large, the incidence of calculous cholecystitis is very high, patient compliance is not consistent, and many patients are willing to undergo surgery after multiple episodes. Therefore, the number of cases leading to direct/early surgery in the acute phase is small and scattered in various medical centers. Most patients are willing to choose delayed surgery after the acute phase of antibiotic support treatment or the more minimally invasive PTGBD drainage in the acute phase and then decide whether to undergo a delayed surgery. According to the actual situation of our center, the management of acute cholecystitis was retrospectively analyzed. PTGBD has a desirable effect on relieving pain and inflammation in the acute phase of patients and remains as the first choice for relieving symptoms in the acute phase of high-risk patients who cannot tolerate emergency surgery. Nevertheless, our results raise the concern that surgery after PTGBD may be more difficult owing to the drainage tube insertion.

This study used the difficulty score for intraoperative findings from TG18 to compare the level of difficulty in performing DLC between the PTGBD and no-PTGBD groups. Traditional indicators, including operation time, blood loss, conversion rate, and morbidity rate, were not sufficiently objective. These variables are easily influenced by the surgeon’s experience and proficiency. The difficulty score for intraoperative findings is desirable as a direct and objective indicator capable of measuring surgical difficulty. We reviewed the videos to assess the difficulty of each DLC surgery procedure using this scoring system to obtain more objective results. In the future, this scoring system can optimally utilize real-time information for prospective studies.

This study had a few limitations. First, there was a statistical difference in age between the two groups, which may have biased the results. In addition, this study is a retrospective analysis, which has certain inherent limitations. Future prospective studies are warranted to confirm our results.

## Conclusions

Our study showed that the operative difficulty of DLC following PTGBD was significantly higher than that in no-PTGBD cases. The PTGBD group showed a significantly longer postoperative stay and operation time, more blood loss, and a higher morbidity rate than the no-PTGBD group. PTGBD is an efficient way to relieve the symptoms of acute cholecystitis. however, it may increase the difficulty and complications of DLC.

## Data Availability

The datasets analyzed during the current study are available from the corresponding author on reasonable request.

## Abbreviations

AC: Acute cholecystitis
DLC: Delayed laparoscopic cholecystectomy
PTGBD: Percutaneous transhepatic gallbladder drainage
